# Microfabricated Electrochemical Cell-Based Biosensors for Analysis of Living Cells *In Vitro*

**DOI:** 10.3390/bios2020127

**Published:** 2012-04-25

**Authors:** Jun Wang, Chengxiong Wu, Ning Hu, Jie Zhou, Liping Du, Ping Wang

**Affiliations:** Biosensor National Special Lab, Key Lab for Biomedical Engineering of Ministry of Education, Department of Biomedical Engineering, Zheda Road No. 38, Zhejiang University, Hangzhou 310027, China; E-Mail: wangjun-47@163.com (J.W.); yebear@zju.edu.cn (C.W.); zjubmehuning@gmail.com (N.H.); zhoujiecomeon@163.com (J.Z.); dlp032506@hotmail.com (L.D.)

**Keywords:** electrochemical biosensor, cell-based biosensor, microfabrication, *in vitro* monitoring

## Abstract

Cellular biochemical parameters can be used to reveal the physiological and functional information of various cells. Due to demonstrated high accuracy and non-invasiveness, electrochemical detection methods have been used for cell-based investigation. When combined with improved biosensor design and advanced measurement systems, the on-line biochemical analysis of living cells *in vitro* has been applied for biological mechanism study, drug screening and even environmental monitoring. In recent decades, new types of miniaturized electrochemical biosensor are emerging with the development of microfabrication technology. This review aims to give an overview of the microfabricated electrochemical cell-based biosensors, such as microelectrode arrays (MEA), the electric cell-substrate impedance sensing (ECIS) technique, and the light addressable potentiometric sensor (LAPS). The details in their working principles, measurement systems, and applications in cell monitoring are covered. Driven by the need for high throughput and multi-parameter detection proposed by biomedicine, the development trends of electrochemical cell-based biosensors are also introduced, including newly developed integrated biosensors, and the application of nanotechnology and microfluidic technology.

## 1. Introduction

A living cell can be properly described as an electrochemical dynamic system [1]. Due to various reduction-oxidation (redox) reactions and changes of ionic composition and concentration [2] in biological processes, cellular life activities are accompanied with electron generation and charge transfer, which can be exploited using electrochemical methods to reveal information about changes in cell function as well as cell growth and development. In this case, cell biochemical parameters, such as concentrations of inorganic ions (H^+^, K^+^, Na^+^, Ca^2+^, Cl^−^, *etc*.), morphological change, membrane potentials and redox potentials, can be detected electrochemically. 

Cell-based biosensors on the basis of electrochemical detection can realize cell analysis and evaluation through measurement of electrochemical signals such as current, potential, impedance, conductivity, capacitance, *etc*., and have become a focus of research in the field of biosensor. In recent years, with the development of nanotechnology and enhancement of interface between sensors and cells, a series of electrochemical cell-based biosensors is used in the study of cell type, concentration, viability, proliferation, apoptosis and molecule distribution inside and outside cells [3,4,5,6,7,8,9,10,11,12,13,14]. From another point of view, cell-based biosensors can employ immobilized living cells as sensing elements, and the specific reaction between stimulus and cells can reveal the intracellular and extracellular microenvironment condition, which can be applied in drug screening and even environmental monitoring [15]. 

In recent decades, the development of microfabrication technology and micro-electromechanical systems (MEMS) have helped to materialize the miniaturization of cell-based biosensors. The most typical is the development of MEA, which has become a powerful tool in research on bioelectric potential [15] since first reported by Thomas [16] in 1972. Recently, there have been new developments of using microfabricated electrode array systems and stimuli-responsive biomaterials to immobilize viable cells *in situ* [17,18]. The ECIS technique [19], which has matured in cell morphology study [20], is greatly promoted by the microfabrication technology, and thus diversification of electrode design is facilitated [21,22,23]. Semiconductor technology stimulates the development of new cell-semiconductor hybrid biosensor systems, such as the ion-selective field effect transistor (ISFET) [24] based on the properties of the electrolyte insulator semiconductor (EIS) system, and another type of promising field effect transistor utilizing the electrolyte-semiconductor interface for achieving biosensing [25,26]. Among these, LAPS [27], based on the photovoltage technique, received extensive attention because of its good sensitivity, stability and high signal-to-noise ratio. Using LAPS, the response of cells to chemical substances is studied by monitoring the acidification of living cells [28] and changes in concentration of other inorganic ions [29]. These miniaturized cell-based biosensor systems are capable of real-time, noninvasive, label-free measurements, which guarantees the potential in on-line biochemical analysis of living cells *in vitro* and facilitates the development of new analytical instruments based on these biosensors. 

Here, we start with the presentation of principles of biochemical cell-based biosensors, including MEA, ECIS and LAPS. Then, their applications in biochemical monitoring of living cells are introduced combined with descriptions of MEMS technology and photovoltage technology. Finally, we survey the developing trends of biochemical cell-based biosensors, including the integration and multifunction requirements, combined with hot topics about microfluidic technology and nanotechnology. 

## 2. Principles of Electrochemical Cell-Based Biosensors

### 2.1. Theory and Structure of Microelectrode Array

MEA is an electrochemical biosensor developed to detect the action potential (AP) in the extracellular microenvironment of cells. On an MEA, a thin metallic film is fabricated between a substrate of glass or silicon and a passivation layer with several electrode sites exposed for sensing the extracellular field potential changes generated by the objective cells. 

When spreading on the microelectrodes, cultured cells adhere to the substrate. But there is still a minute volume of electrolyte between the cells and the microelectrodes; thus, a solid-liquid interface on the electrode surfaces is formed. The electrochemical properties of the interface are the basis of the sensing mechanism of MEA. 

According to the electric double layer (EDL) theory, when a metal is placed into ionic liquid, an equilibrium condition is established once the charge transfer between the metal and the solution is equal. The electric field on the interface generated by electron transfer causes the formation of an inner Helmholtz plane (IHP) and an outer Helmholtz plane (OHP). The net reaction induces the creation of an electric double layer, which is also an electrified interface describing the interphase region at the electrolyte boundary [30]. 

**Figure 1:**
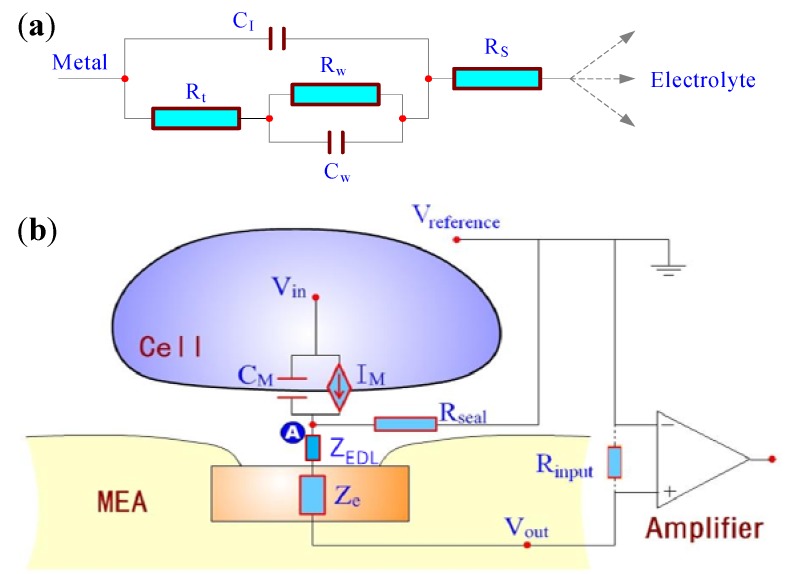
(**a**) The equivalent circuit of metal-electrolyte interface; (**b**) The equivalent circuit of the signal pathway in MEA system. V_in_: the intracellular potential; C_M_: the capacity of the cellular membrane; I_M_: the current source of the cellular membrane; A: the junction between the cell and electrode; R_seal_: the sealing resistance between the cell and chip; R_input_: the equivalent of the input of preamplifier; V_out_: the output potential of the microelectrode; V_reference_: the grounded bulk media potential.

The equivalent circuit of metal-electrolyte interface can be explained with the Randles model, as shown in Figure 1(a). In the circuit, an interfacial capacitance (C_I_) is in parallel with charge transfer resistance (R_t_) and diffusion related Warburg element (R_W_ and C_W_). The spreading resistance (R_S_) represents the effect of current spreading from the localized electrode to a distant counter electrode. 

An equivalent circuit (Figure 1(b)) of the signaling pathway illustrates how a biological signal is converted into an electrical one [31]. There are mainly three components in the equivalent circuit. Firstly, the transmembrane potential functions as an electric source (V_in_). Secondly, the parallel circuit of C_M_ and I_M_ expresses an overall effect of the cellular double-lipid layer structure, ion channels and cellular signal pathway. Finally, the electrolyte between the cells and the electrodes induces part of the current to leak out to the bulk medium. This effect is expressed as the sealing resistance (R_seal_). The current flowing through the basal membrane is the sum of the ionic current (I_ionic_) and the capacitive current (I_M_). Thus, the total transmembrane current is given by: 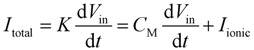
(1)

**Figure 2:**
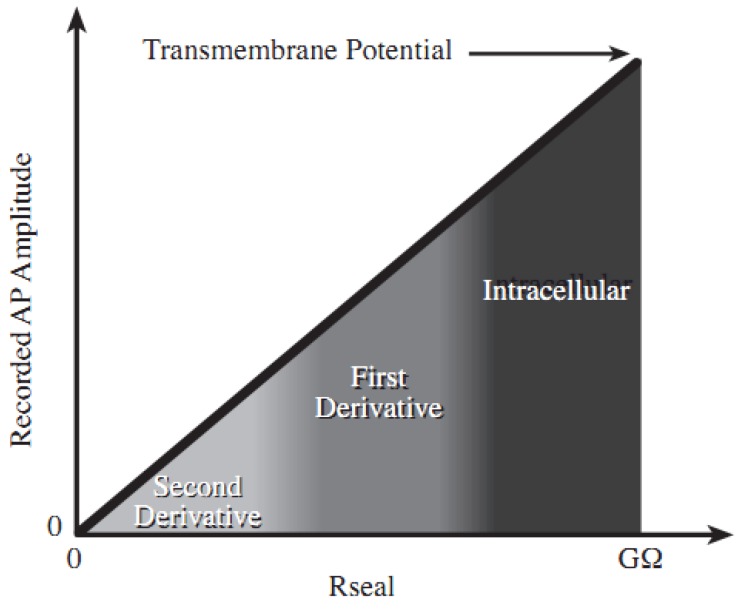
Characteristic AP signal recorded with an extracellular microelectrode as the seal resistance is varied. At low R_seal_, the amplitude is small and a second derivative behavior is observed (assuming no additional derivative is generated due to the electrode itself). As R_seal_ increases, the amplitude of the recorded AP signal increases and the order of the derivative decreases. For extremely high R_seal_, a whole-cell patch configuration is approached and the intracellular signal is measured.

Accordingly, the voltage at node A is proportional to the second derivative of the transmembrane potential. This voltage is also proportional to the magnitude of R_seal_ [32]. The larger R_seal_ is, the better the recorded signal can reflect the transmembrane potential. In the extreme condition of an infinite seal resistance, the voltage at node A would correspond to the intracellular potential, thereby simulating a whole-cell patch configuration. This trend is shown in Figure 2 [32,33,34].

In MEA design, the ‘sandwich’ structure has been employed since it was first pioneered by Thomas in 1972 [16]. Firstly, a thin metallic layer is deposited on an insulative substrate as the sensing layer. Then a passivation layer is coated on it with electrode sites and pads exposed for electric sensing and signal output. 

**Figure 3:**
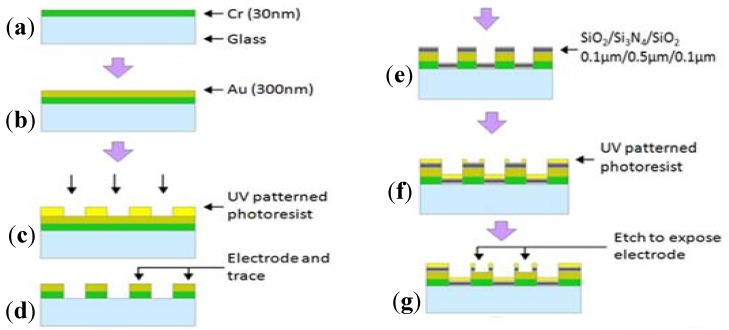
General fabrication process of MEA.

The flow chart in Figure 3 is a general fabrication process of glass-based MEA. In the beginning, Cr (30 nm) and Au (300 nm) are sequentially sputtered or evaporated onto the glass substrate. The use of the Cr layer can enhance the adhesion of the Au layer onto the substrate. Then the electrodes and traces are patterned from this composited metallic layer by conventional lithography and etching techniques. After that, an insulative layer of SiO_2_ is deposited onto the chip surface by plasma-enhanced chemical vapor deposition (PECVD). Finally, the electrodes and pads are exposed from the silicon nitride layer by reactive ion etching. The fabrication of 3D MEA shares the design idea of planar MEA [35,36]. 

**Figure 4:**
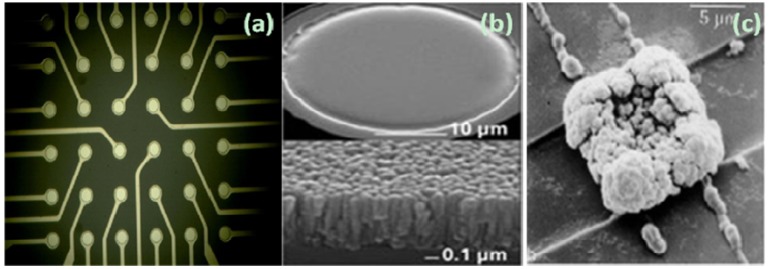
The surface morphology of microelectrodes with different treatment. (**a**) The chip layout of a 6 × 6 microelectrode array; (**b**) A microelectrode sputtered with T_i_N (http://www.multichannelsystems.com/); (**c**) A microelectrode electroplated with platinum black. (Reprinted from [38]. © 1980, with permission from Elsevier).

Figure 4 shows a typical fabricated MEA chip. The electrode is designed to match the size of a cell, with a diameter of 10–100 μm. To reduce the electric interference between electrodes, the center-to-center distance is usually more than 100 μm. To lower the impedance and improve the performance of the electrodes, usually a T_i_N or platinum black layer is deposited on electrodes [37]. 

### 2.2. Electric Cell-Substrate Impedance Sensor

**Figure 5:**
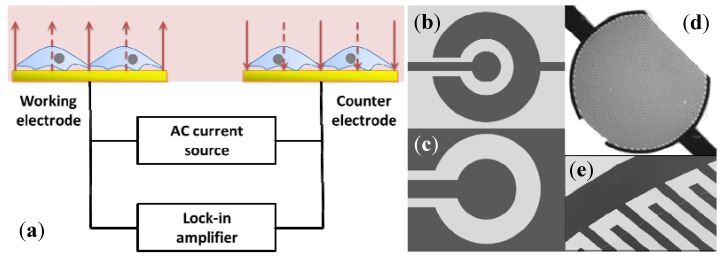
(**a**) Schematic of the measurement setup; (**b**,**c**) Monopolar electrodes: a small electrode is used as the working electrode and a larger electrode as the counter electrode. Since the ratio of the surface area of the two electrodes is over a couple of hundred, the total impedance of the system is dominated by the impedance change at the small measuring electrode; (**d**) Interdigitated electrodes (IDEs): a plurality of independently-operating IDE units connect to a terminal strip to form one branch of an electrode. The other identical one is parallel spaced; (**e**) Interdigitated electrode array with parallel lines.

Before growth and propagation, cell adherence to substrate or surface is always the first step in a cell-based experiment. ECIS is one of the electrochemical techniques that can be applied to monitor cell adhesion, spreading, and motility in real time [39]. Figure 5 shows a schematic overview of the measurement setup pioneered by Giaever and Keese [40]. In this setup, microelectrodes are constructed beneath the cell attachment platform. An alternating current is applied on the electrodes and the voltage is monitored using a lock-in amplifier. If no cells are cultured on the surface, electric current can flow freely from the surface to the electrodes. Cells growing on the electrode will impede the current flow and thus increase the resistance of the system (measured in ohms) because cell membrane acts as an insulator. The magnitude and time course of the increase is found to become more pronounced when the cell density during seeding approaches confluence. Electrodes without any cells will produce a minimal ‘base-line’ resistance. The addition of analytes that either rupture the cell monolayer or cause disturbances will lead to changes in impedance. 

Typically, two structures are used in impedance sensing: the monopolar electrode [41,42] and the IDEs [21,43]. With monopolar structure, the area excluding the working electrode is relatively large, only a few cells on the working electrode contribute to the measured impedance, which results in fluctuations among experiments. Moreover, large counter electrode hinders further miniaturization, which is essential in a high-throughput screening (HTS) system. In contrast, the interdigitated structure shows improved performance as the current flows in the vicinity of the electrodes surface. A much higher sensitivity to impedance change is demonstrated compared with the conventional design. In addition, the IDEs commonly cover up to about 50% of the area of each well, lowering the well-to-well differences in the HTS system. However, no preference can be given to these two prototypes without specific applications. 

Generally, the fabrication process of ECIS is the same as MEA, with a metallic layer deposition on the substrate and a following lithography and etching process, as shown in Figure 3. 

### 2.3. Light Addressable Potentiometric Sensor

LAPS is a semiconductor device proposed by Hafeman *et al.* in 1988 [27]. With a structure of Si/SiO_2_/Si_3_N_4_, it can be excited by a modulated light source to generate a photocurrent which corresponds to the potential on the Si_3_N_4_ surface. In principle, any effect that results in the change of surface potential can be detected by LAPS, including the ionic change [44], redox reaction [45], *etc*. The fabrication process of LAPS is easier due to the simple structure, and the encapsulation of LAPS is much less critical. Besides, the extremely flat surface makes it convenient to incorporate into the micro-volume chamber, which facilitates trace measurement. Therefore, LAPS holds promise as a preferred platform for future cell-based biosensors. 

**Figure 6:**
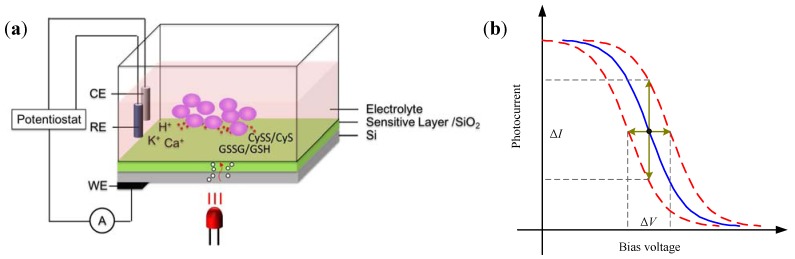
The schematic diagram of LAPS as a cell-based biosensor. (**a**) Working principle of LAPS; (**b**) Characteristic *S*-shaped curves.

LAPS is a potentiometric semiconductor device that could have an EIS structure or an electrolyte-metal-insulator-semiconductor (EMIS) structure, as shown in Figure 6. Generally, it has the layer sequence of an Si/SiO_2_/sensitive layer. An external DC bias voltage is applied to form an accumulation layer, a depletion layer or an inversion layer at the interface of insulator (SiO_2_) and semiconductor (Si). When an AC modulated light illuminates the LAPS chip, the semiconductor absorbs energy, leads to energy band transition and produces electron-hole pairs. Electron and hole would combine soon and photocurrent cannot be detected by peripheral circuit without light illumination. When LAPS is biased in depletion, an internal electric field is produced across the depletion layer, and the width of the depletion layer is a function of the local surface potential. When a modulated light illuminates the bulk silicon, light induced charge carriers are separated by the internal electric field and thus photocurrent can be detected by the peripheral circuit. The amplitude of the photocurrent depends on the local surface potential. Therefore, by detecting the photocurrent of LAPS, local surface potential can be obtained [27]. 

When cells change the surface potential of LAPS through the ions (H^+^, Na^+^, K^+^, Ca^2+^, *etc*.) or redox couples like CySS/CyS (cystine/cysteine) and GSSG/GSH (oxidized/reduced glutathione), the actual bias voltage varies, and fluctuations are generated in the corresponding photocurrent (Figure 6(a)). Therefore, by focusing the light spot on the surface cultured with cells, ionic changes or redox potential can be detected through the local surface potential measurement. Thus LAPS is applied in cellular metabolism detection, cytotoxicity evaluation and drug analysis. 

**Figure 7:**
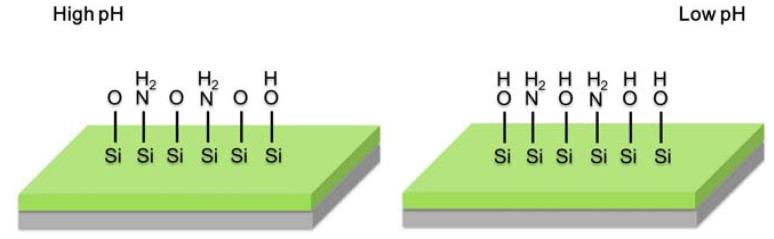
The formation state of silanol (Si-OH) and silamine (Si-NH_2_) groups both in high and low pH conditions.

For pH detection, a layer of Si_3_N_4_ is fabricated on the surface of LAPS. This thin layer of silicon oxynitride is used as the insulating layer that can effectively separate the silicon substrate from the electrolyte. According to the site-binding theory [30,46] which is also demonstrated in the semiconductor sensor based on field-effect [47,48], a potential difference related to H^+^ concentration of the electrolyte arises on the insulator (Si_3_N_4_/SiO_2_)-solution interface. The insulating layer interacts with protons in the solution to form groups of silanol (Si-OH) and silamine (Si-NH_2_). Therefore, the sensor surface potential is determined by the protons in the solution. As is shown in Figure 7, if a voltage is applied to the sensor, an electric field is formed at the silicon-insulator interface. By illuminating the rear side of the sensor, a photocurrent is generated [49]. This photocurrent corresponds to a hole-electron pair created by radiation absorption at an atomic level. Since a field potential is formed in the chip, holes and electrons move in opposite directions, resulting in the creation of local current which can be output from the backside aluminum layer. 

The amplitude of the photocurrent depends on the applied bias voltage and the sensor surface potential determined by the pH value of the electrolyte. The insulator surface is partially covered by silanol and silamine, both of which can be considered as a function of pH. The surface is neutral around pH 3.5. Therefore, under physiological conditions (pH 7.4), the surface becomes negatively charged, which makes the surface potential pH-dependent. Such a dependence is Nernstian and linear from pH 2 to 11 [27]. Generally the characteristic current-voltage curve (I–V curve) is S-shaped (Figure 6(b)). In the continuous measurement, the applied bias voltage is fixed at a constant value, which is selected as the inflection point of the I-V curve. By doing this, the recorded photocurrent change can reflect the pH variation most sensitively. Then the variation of photocurrent Δ*I* represents the pH change. 

Electron exchange accompanies oxidation-reduction reactions, which can be detected by a noble electrode (Pt or Au electrode) through potentiometric measurement. The potential on the electrode (also redox potential) is determined by the ratio of the concentrations of the solution species [50] and the value is stated by the Nernst equation. Redox potential measurements can also be obtained by depositing a metal layer (often Au) on the insulator surface of the EIS structured sensor. When in contact with an aqueous solution which contains a redox pair, the surface potential of the metal layer reflects the ratio of the redox pair’s concentration. The Au layer deposited functions as an electron transfer electrode similar to a traditional Au electrode, and the equilibrium potential formed on the surface of the layer is measured by photovoltage technology. As the EMIS structured sensor and EIS structured sensor have similar structure, fabrication and measurement technology, they can share the same measuring system. 

**Figure 8:**
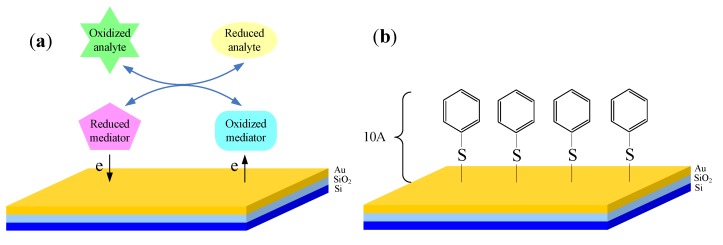
Schematic diagram of the interface between biological analyte and EMIS structured sensor. (**a**) Redox couples functioning as mediators used to be added into samples, which usually can be Fe(III)/Fe(II) or quinhydrone; (**b**) Modified interface using electron transport promoter to accelerate electron transport between analyte and sensor surface, here 4-PySH is taken as an example.

It is worth mentioning that measurements of reversible, Nernst potentials are accompanied by a number of requirements, among which the most important is the need of fast electron exchange between the metal layer and the analyte. Slow electron transfer is not propitious to the formation of stable equilibrium potential, and this is responsible for the failure of potentiometric measurements of biological samples. To obtain meaningful analytic information, mediators are usually used so that stable redox potential measurements could be made. The mediators can be redox couples directly added to the samples, like Fe(III)/Fe(II) [51] or electron transport promoter, such as 4-pydidinethiol (4-PySH) and bis(4-pyridyl)disulfide [52] that are to be modified on the metal surface (Figure 8). 

**Figure 9:**
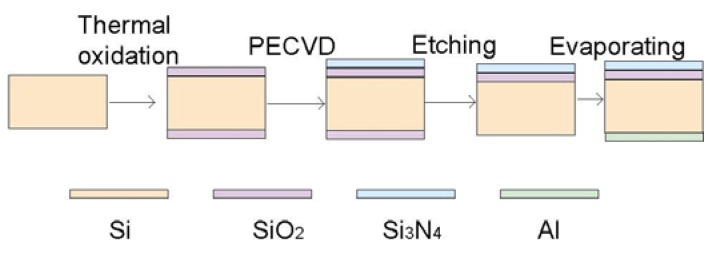
Fabrication process of LAPS chip: thermal oxidation, PECVD, etching, evaporating.

The LAPS device usually consists of the heterostructure of Si/SiO_2_/Si_3_N_4_. Fabrication of LAPS is easy and fully compatible with the standard microelectronics facilities (Figure 9). A silicon chip is chosen as the substrate with a resistance of about 5–10 Ω∙cm. Then a SiO_2_ layer is formed with a thickness of 30–50 nm. A 50–100 nm layer of Si_3_N_4_ was deposited by PECVD as a sensitive layer on the upper side of the bulk. For the EMIS structured sensor, a deposition step of 30 nm Cr and 100 nm Au on the Si_3_N_4_ surface is necessary. Finally, an aluminum membrane about 300 nm in thickness is evaporated on the backside of the silicon chip to form an ohmic contact. 

## 3. Applications in Biochemical Monitoring of Living Cells

### 3.1. Cellular Electrophysiology Detection

MEA is a noninvasive and long-term method enabling stimulation and recording of bioelectricity with high spatial and temporal resolution in cell and tissue cultures. As the MEA technology can be applied to any electrogenic tissue or cell, that is, central and peripheral neurons, cardiomyocytes, and retinas, the MEA biosensor is an ideal *in vitro* system to study the dynamics and physiology of these cells or tissues and to monitor both acute and chronic effects of drugs and toxins. 

#### 3.1.1. Cardiomyocyte Detection

**Figure 10:**
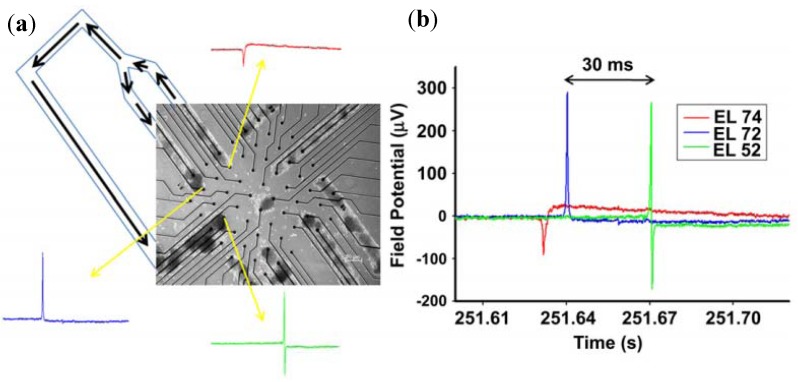
AP conduction in patterned cardiac myocyte monolayers. (**a**) Phase contrast picture of cardiac myocyte patterns on the top of the substrate embedded electrodes (electrode distance is 200 μm) with a sketch of the conduction pathway on the completed pattern and with the recorded extracellular signals on the given electrodes; (**b**) Overlay of the recorded traces showing the temporal relationship of the signals. (Reprinted from [53]. © 2011, with permission from Elsevier).

The cardiac biomarker sensing [26] using MEA has been reported. In addition, the cultured cardiomyocytes form a confluent contracting layer when mature and they exhibit spontaneous, rhythmic and synchronous excitability. The physiology of cardiomyocytes such as propagation and conduction properties is also learnt by the MEA cardiac biosensor. Natarajan *et al.* [53] developed a modified MEA for cell patterning to study the cardiac physiology. Self-assembled monolayers (SAMs) were modified on the surface of MEA with a photolithography method. An adsorbed fibronectin layer was used as the foreground to support cardiac myocytes attachment and growth, and a poly (ethylene glycol) (PEG) SAM was used as the background to prevent protein adsorption. By doing this, the beating cells were allowed to grow exclusively over specific electrodes. With patterning of the cells on the electrode array, the exact path of a spontaneous excitation wave can be determined and then, using the path length, conduction velocity can be calculated with a high degree of accuracy. The conduction velocity was 0.190–0.025 m/s for spontaneous firing of the patterned cardiomyocytes over eight different MEAs (Figure 10). The conduction velocity at stimulation mode under different frequencies was also measured by this system. This helped to understand the electrophysiological properties of cultured cardiomyocytes. A device for separated and reversible co-culture of cardiomyocytes provides a meaningful platform for studying the AP propagation between cardiomyocytes and skeletal myoblasts through the analysis of conduction velocity across the MEA [54]. 

#### 3.1.2. Brain Slice Detection

**Figure 11:**
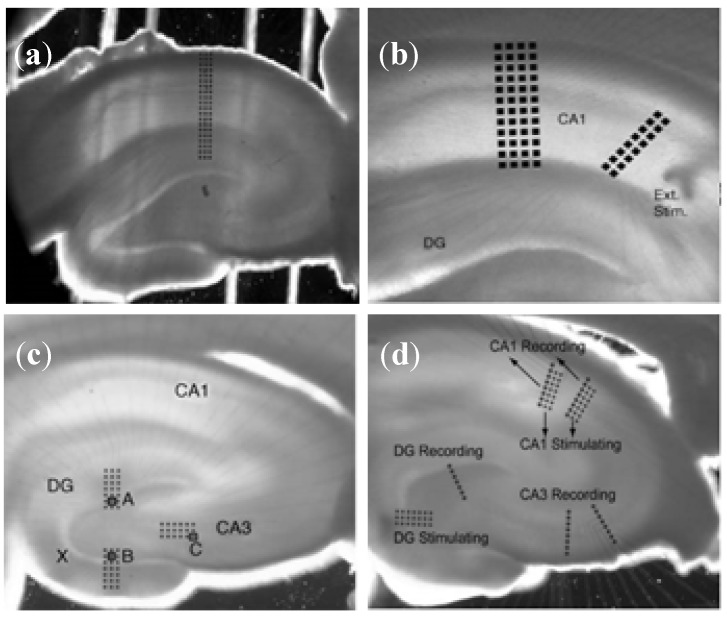
Conformal probes. (**a**) cMEA#1 is a 3 × 20 rectangular array well suited for electrophysiological investigations of the pyramidal and granular cells of hippocampus; (**b**) Using cMEA#2, monosynaptic input/output (IO) curves were recorded in CA1 in response to SchC stimulation; (**c**) Monosynaptic and disynaptic responses were recorded from DG and CA3 respectively by stimulating (perforant path) PP with external electrodes; (**d**) The cMEA#4 was designed to record the monosynaptic response of the DG, disynaptic response in the CA3 area and trisynaptic response in the CA1 when the PP is stimulated. (Reprinted from [55]. © 2006, with permission from Elsevier).

Compared with the dissociated cultures, slices hold the closer characteristics to that of the intact tissues. Hippocampal slice is the optimal object to study excitatory and inhibitory transmission as well as synaptic plasticity. Microelectrodes are designed in a tissue-conformal and partially high-density distribution for specific stimulation and recording experiments in acute hippocampal slices [55]. Four custom-designed planar MEAs (cMEAs) in different configurations which conform in design to the slice cytoarchitecture are well suited for specific electrophysiological applications (Figure 11). The high-density provides high spatial resolution for selective stimulation of afferent pathways (Schaffer collaterals; medial *versus* lateral perforant path) and recording of the corresponding responses. It also enables current source density (CSD) analysis of laminar profiles obtained through sequential stimulation along the CA1 pyramidal tree.

MEAs are also feasible for studying neuronal regeneration between different tissues and the effect of drugs [56,57]. The 3D-structure MEAs can achieve a better tissue-adhesive surface to increase electrical recording and stimulation performance. 

**Figure 12:**
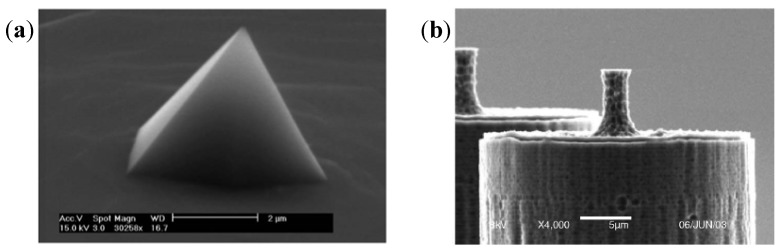
(**a**) SEM of cone-shaped electrodes on glass substrate. (Reprinted from [36]. ©2006, with permission from Elsevier) (**b**) SEM of cylindrical electrodes on silicon substrate. (Reprinted from [61]. © 2006, with permission from Elsevier).

High-density three-dimensional carbon nanotube (CNT) can be coated on MEAs to create extremely rough surfaces and this is effective in interfacing with slice or supporting neuronal adhesion. Thus a remarkable signal-to-noise ratio can be obtained [58]. Based on the deep reactive ion etching (DRIE) technique for silicon substrate, Charvet *et al.* [59] developed high-density arrays of 3D microelectrodes, which offered new possibilities for *in vitro* studies of acute brain slice. Rajaraman *et al.* [60] fabricated solid or hollow towers at a height up to 500 μm above a substrate with metal lines defined on them. The towers targeted specifically electrophysiological measurements of slices or large neural network. Figure 12 shows two typical 3D electrodes with cone-shape or cylindrical tips. However, the hard contact between slice or cells and a bed of needles or pillars may have some unpredictable effects on cell membranes and functions. 

All experiments and results above indicate that MEA is feasible for acute brain slice experiments and effective for neural networks. 

#### 3.1.3. Dissociated Neural Network Detection

**Figure 13:**
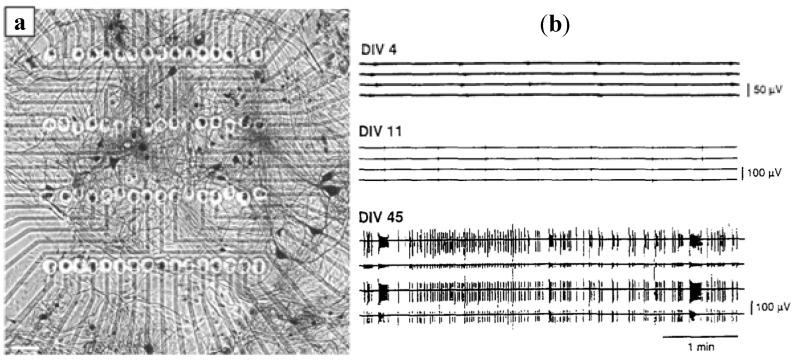
(**a**) Neuronal network derived from murine spinal cord tissue (92 days *in vitro*), grown on the recording matrix of a 64-electrode array plate. (Reprinted from [70]. ©2007, with permission from Elsevier) (**b**) Developmental changes in neuronal activity. Random bursts were observed at DIV4, tightly synchronized activity appeared at DIV11. The mature neuronal activity consisted of a complicated, high order pattern of spike-like firing and bursting at DIV45. (Reprinted from [71]. © 1996, with permission from Elsevier).

The dissociated neural network from mammalian tissues is an invaluable model to study learning, memory and information processing in the brain. When cultured on MEA, the spike activity from neurons can be recorded at about 5 to 7 days. A consistent bursting activity is likely to be dominant after 18 to 25 days *in vitro* [62], depending on the cell culture system used. Bursting stems from the regulation of the balance between intrinsic excitation and inhibition within the network. It may be related to calcium wave and sodium currents [63,64]. The synaptic connections and network topology play fundamental roles in the bursting process. Figure 13 shows the spinal cord neural networks grown on an MEA chip (Figure 13(a)) and developmental changes in neuronal activity of the cortex neural network (Figure 13(b)). Thus, excellent experimental conditions are provided for studying structural, functional development of cultured neurons and dynamic neural network. 

3D-structure MEAs are also used for strengthening the contact between neural networks and electrodes to obtain a high signal-to-noise ratio. In addition, planar MEAs are designed to guide the neurons’ growth onto the defined electrodes or to guide the neural network to form predetermined patterns. Mostly, well-defined electrode arrays were fabricated on silicon substrate to generate dielectrophoretic (DEP) forces, and the desired number of neurons were immobilized onto recording electrodes [65]. Jing *et al.* [66] used SAM to create hydrophobic and hydrophilic areas on MEAs, which would lead a patterned neuronal network on the substrate. Spontaneous electrical signals were detected from the networks of GT1-7 neurons and primary mouse hippocampal neurons. By using a polymer layer such as polydimethylsiloxane (PDMS) and defined chambers or microfluidic channels, the growth of the cultured neuronal network was guided. Thus, the communication between groups of neuron networks or signal transduction in different cells can be mapped [67,68,69]. 

#### 3.1.4. Other Electrogenic Tissues or Cells Detection

Embryonic stem cells can be induced to differentiate into different cell types which display similar molecular, structural, and functional properties. It may provide a possible solution for the lack of *in vitro* human cardiac tissue models for a variety of applications. MEA can be used to record the electrophysiology activity of stem cell-derived electrogenic cells such as neurons and cardiomyocytes [72,73]. The combined system could be a high-throughput platform for detecting and analyzing any desired type of toxicant or pathogenic threat. 

The vertebrate retina, an easily accessible part of the central nervous system, is a light-sensitive part located in the inner layer of the eye. It is composed of three layers of nerve cell bodies and two layers of synapses. Light stimulation results in a complex signaling of neurons within the layers of the retina. The ganglion cells, a type of neuron located near the inner surface of retina, transmit visual information from retinal photoreceptors to the brain nervous system. When light impulses fall on the retina, trans-retinal voltage change due to the activation of potassium channel on neurons and Müller glia cells, and the changes are measured as the electroretinogram (ERG) [74]. 

**Figure 14:**
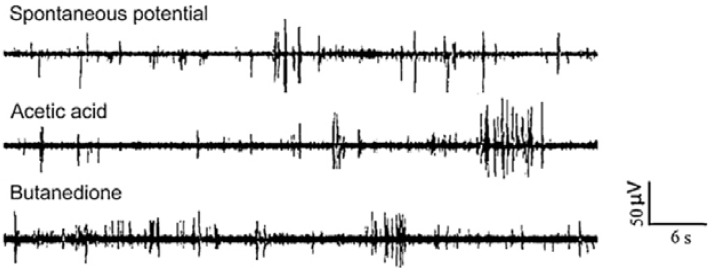
Electrophysiological signal changes of olfactory cells after stimulation of acetic acid and butanedione. (Reprinted from [75]. © 2010, with permission from Elsevier).

MEA is also a stable platform for detecting electrophysiological activities of olfactory and gustatory system. The biological olfactory system has high sensitivity and specificity to discriminate different odors. In the work of Liu *et al.* [75], the stripped olfactory epithelium was fixed on the surface of a 36-channel MEA. Then, the extracellular potentials of the olfactory receptor neurons in the epithelium were recorded after stimulation of odorants such as acetic acid and butanedione. The olfactory cells generated firing with different modes (Figure 14). Through time-domain and frequency-domain analysis, the firing characteristics were found to be different from that of spontaneous potentials. The detection principle of gustatory cell-based MEA was similar. When exposed to substances with different tastes, such as sodium chloride, quinine and sodium glutamate, the recorded potentials represented different firing modes according to the power spectrum analysis. The multi-channel signal analysis has potentials in revealing some spatial and temporal information of early olfactory/gustatory sensing for bioelectronic nose/tongue.

### 3.2. Cellular Morphology Detection

#### 3.2.1. Cell Adhesion and Proliferation

When a cell in suspension encounters a solid surface, a series of cellular morphology changes arise until a complete adhesion is formed between the cell and the substrate [76,77]. The phenomenon of cell adhesion and spreading on the substrate can be detected using ECIS technology; thus, the underlying interactions of cells and substrates can be figured out, which can be used to promote cell culture quality and cell assays *in vitro*. 

Cell adhesion and proliferation depend strongly on the interactions between cells and extra-cellular matrix (ECM) substrates. Although the traditional detecting methods such as WST-1 assay, XTT/MTT assay, BrdU assay, fluorescence microscopy and force microscope are capable of exploring cell-substrate interactions quantitatively, they are usually laborious, indirect and not time-resolved. The impedance sensing provides a solution for the revelation of the kinetics of cell adhesion and proliferation. Efforts using ECIS to study cell-ECM interactions are mainly divided into two categories. In the first case a certain type of cells adhere to a specific adhesive substrate, modified by various ECMs, such as fibronectin, collagens, laminins, poly-L-Lysine and vitronectin [78,79]. In the other case, specific reagents are used to block the cell-ECM interaction or to disrupt the cytoskeletal architecture or cell-signaling pathways, including G-protein coupled receptor activation and protein tyrosine kinase activation [80,81,82]. 

After cell adhesion, ECIS can be effectively used in the study of cell proliferation progression, revelation of mechanisms underlying cell cycle [83,84,85], identification and quality control of cell differentiation [86,87,88], and characterization of tissue cultures [89,90,91,92,93,94]. In comparison with conventional methods such as autoradiography, fluorescent staining and flow cytometry, the advantages of ECIS are real-time, label-free, non-invasive monitoring, and easy-construction. Moreover, an *in vivo* environment can be mimicked in an ECIS system *in vitro*, which is essential in various biomedical applications such as toxicological analysis, high-throughput drug screening, stem cell research, and tissue engineering. 

**Figure 15:**
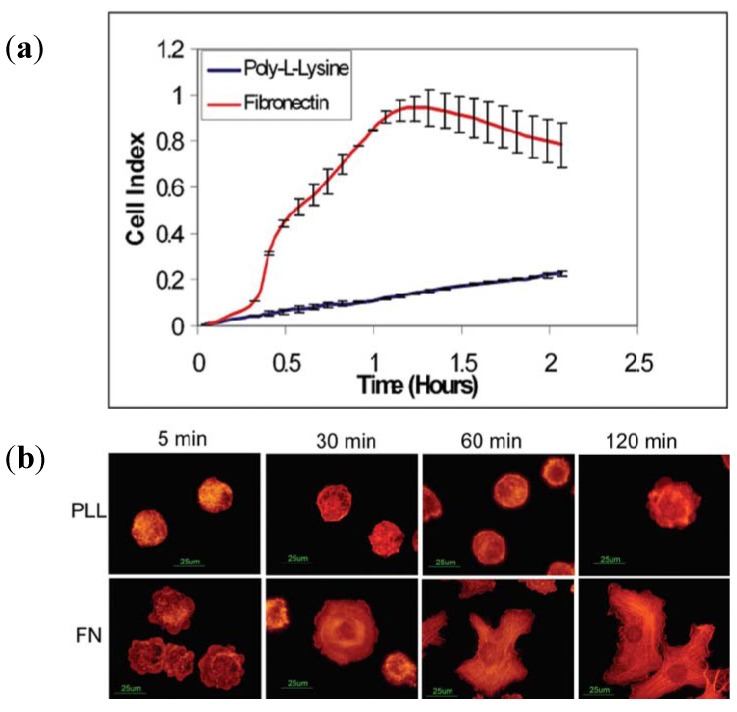
(**a**) Attachment and spreading of NIH3T3 cells on FN and PLL coated ECIS sensors monitored by RT-CES system. The cell index was displayed with an arbitrary unit. Ten thousand cells were seeded per well of E-plate in triplicate. The experiment was carried out at least 6 times; (**b**) Attachment and spreading of NIH3T3 cells on FN- and PLL-coated chamber slides. (Reprinted from [95]. © 2005, with permission from SAGE Publications).

Figure 15 displays a typical investigation of cell adhesion and proliferation on different ECM-coated surfaces. NIH3T3 cells were cultured in the wells of ACEA E-plates, and the chamber coated with fibronectin (FN) or poly-L-lysine (PLL) was used as a control. At the same time, chamber slides were also coated with FN or PLL and the same numbers of cells were added to each well. The dynamic monitoring of cell-substrate impedance change due to cell adhesion and proliferation was completed by RT-CES system (by ACEA Biotech Inc.), while the real state of cells was determined by staining with rhodamine-phalloidin and visualized using an epifluorescent microscope. Figure 15(a) shows the relationship between cell-substrate impedance and the physiological state of cells (mainly cell number and morphology), while Figure 15(b) presents the quality of cultured cells on various ECMs. To determine the concentration-dependent effect of coated FN on the extent of cell adhesion and spreading, E-plates were coated with increasing concentrations of FN ranging from 0 to 20 µg/mL. Further results were presented in the literature [95]. 

#### 3.2.2. Cell Migration and Metastasis

Cell migration plays an essential role in various complex processes such as embryonic development, homeostasis, immune response, wound healing, and cancer metastasis. Up to now migration has become the focus of many studies [96]. 

**Figure 16:**
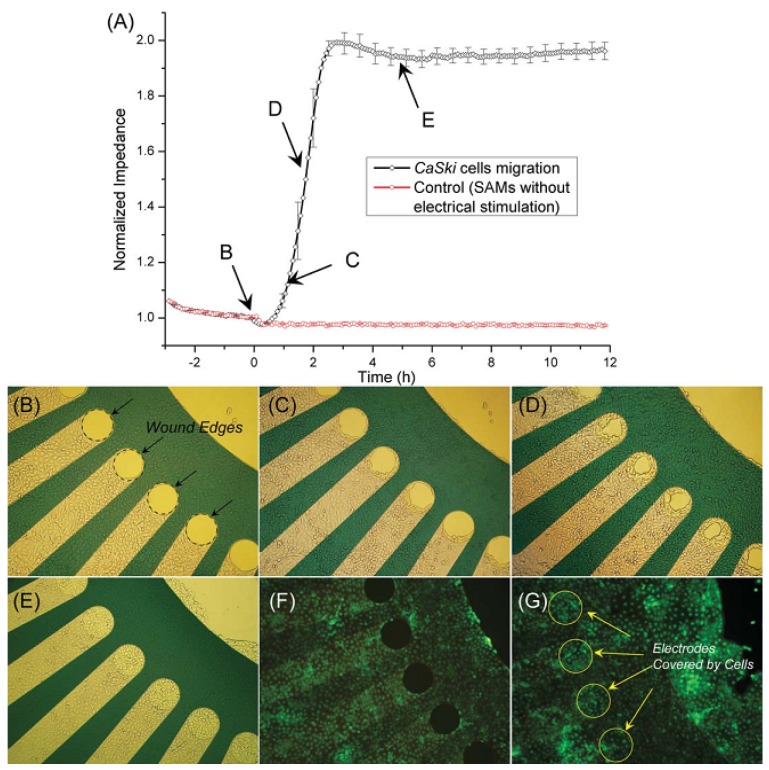
(**a**) The impedance variation for CaSki cells showing the real-time progress of cell migration; (**b**–**e**) The photographs taken in the progress of cell migration onto the electrodes; (**f**,**g**) The fluorescence image of the sensor, showing cell viability after modification of the SAMs and application of the DC current (4 times independent repeats for the experimental data). (Reprinted from [42]. © 2012, with permission from the Royal Society of Chemistry).

Wound healing assays have been carried out in tissue culture for many years in the study of cell behavior, including appraising the migration and proliferative capacities of different cells under various culture conditions. The application of impedance sensing in wound-healing investigation was first reported by Noiri *et al.* [97]. They electro-permeated the confluent cell monolayer to generate wounds on the electrode using a DC current. The rate of restitution of monolayer integrity, as judged by the restoration of the electrical impedance, was monitored by an electrical impedance sensor for 20 h. Then, Keese *et al.* [98] improved the wound generation methods by using AC currents in the milliampere range at high frequency, so the wound was restricted to the small electrode and no electrode damage was found. This method provided highly reproducible results comparable to that observed in traditional wound-healing experiments. Wang *et al.* [42] modified the wound healing methods by using SAMs. The SAMs were formed on the electrodes to inhibit cell adhesion, which could effectively mimic wounds in a cell monolayer. After a DC pulse was applied, the SAMs were destroyed and cells began to migrate (Figure 16).

**Figure 17:**
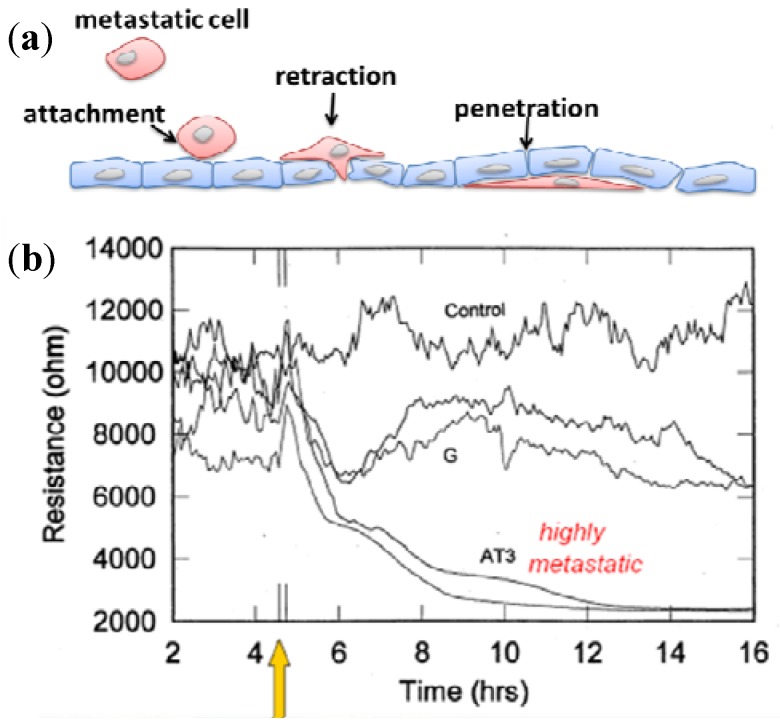
(**a**) Metastatic cells that invade the confluent HUVEC layer and cause damage to the barrier integrity; (**b**) HUVEC layer challenged with the weakly metastatic G subline or the highly metastatic AT3 subline. The loss of resistance was due to the loss of integrity of the endothelial cell layer in response to the activities of the cancer cells.

Migration also occurs during metastasis when some cancer cells migrate out of the initial tumor into the circulation and move to new locations, where they form a secondary tumor. As angiogenesis and invasion of cancer cells from the primary site into the surrounding area are essential for tumor development, assays have been developed to study these processes [99]. The ECIS-based assay used in cancer metastasis was first presented by Keese *et al.* [100], while the previous study was based on microscopic observations, where metastatic cells added over established endothelial cell layers were observed to attach and invade the cell layer. The extent and rate of drop in impedance could be correlated with the metastatic potential of cancer lines tested (Figure 17). For highly metastatic sublines, within an hour after being challenged, the impedance of the confluent human umbilical vein endothelial cells (HUVEC) layer was substantially reduced, while that caused by the weakly metastatic sublines was less pronounced. 

Tumors undergo a series of changes in the formation of metastases. Many genes and factors have been involved in tumor invasion, including adhesion molecules, cytoskeletal proteins and proteases [101]. Efforts have been made to investigate the function of such factors [102,103,104,105,106]. In these papers, the basic experimental settings were similar to that of Keese’s work. The enhancement of migration caused by certain molecules (lysophosphatidic acid (LPA), placenta growth factor (PlGF), *etc*.), receptor (CD44), cytokine (leptin) or proteases (AKT, rho-associated kinase (ROCK), pocal adhesion kinase (FAK), *etc*.) was first observed. As genes in signaling pathways express biochemical substances that can effectively block invasion and metastasis, the effect of pharmacologic inhibitors like JAK/STAT inhibitors and EGFR inhibitors on the signaling pathways was evaluated. Interestingly, the stable expression of certain genes, molecules or proteases was found to have the ability to inhibit tumor metastasis [107,108,109]. 

In comparison with other chemotaxis assays like Boyden chamber assays, ECIS has several advantages: better performance in mimicking *in vivo* events and high sensitivity and real-time monitoring, which makes it a valuable tool in the exploration of biomarkers or therapeutic targets. 

### 3.3. Cellular Metabolism Detection

**Figure 18:**
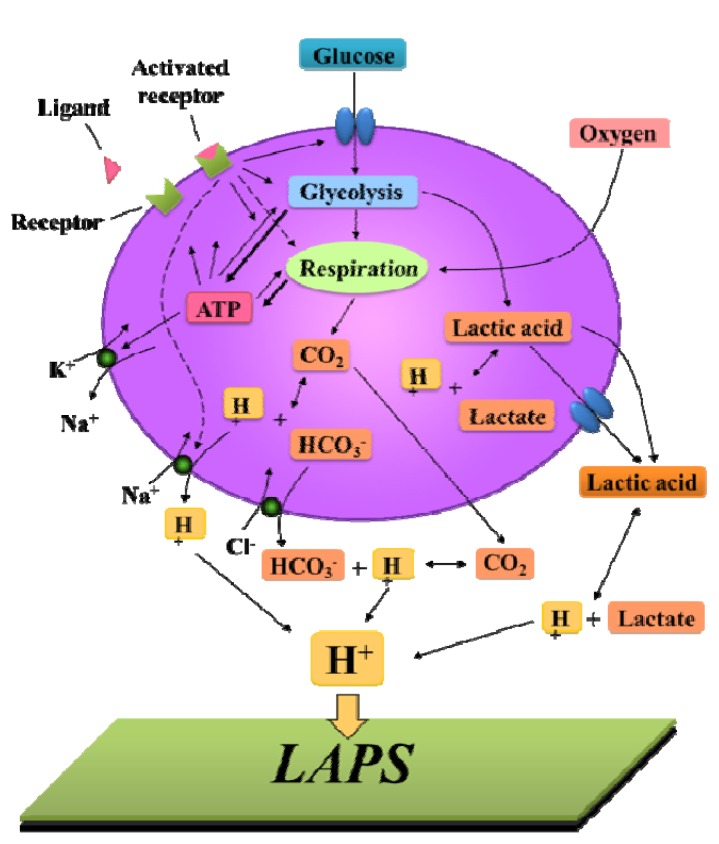
Schematic diagram of extracellular acidification induced by cellular metabolism and physiological processes. With receptor stimulation, the cellular physiological activities will be affected. The corresponding ATP consumption is compensated by the increased uptake and metabolism of glucose, which results in an increased secretion of acidic products. The extracellular acidification can be detected by LAPS. (Reprinted from [111]. © 2012, with permission from World Scientific Publishing Co.).

As the basic physiological feature of life, heterotrophic cells absorb various nutrients, produce energy and secrete acidic wastes for growth and development. Metabolic energy is generated by carbon sources such as sugars, amino acids and fatty acid. The schematic of cellular metabolism and physiological processes is displayed in Figure 18. Under normal conditions, glucose is taken up by the cells and degraded into energy and acidic products. Under natural aerobic conditions, glucose is converted into CO_2_ and energy via glycolysis, the citric acid cycle and oxidative phosphorylation. While under anaerobic conditions, glucose is converted into lactate with energy [110] via glycolysis and combining lactate dehydrogenase. The ATP generation per glucose molecule under aerobic conditions is 19 times higher than that under anaerobic conditions (38 ATP/glucose *versus* 2 ATP/glucose), while the acidic product is much less than anaerobic conditions (0.167 H^+^/ATP *versus* 1 H^+^/ATP). 

Lactic acid and CO_2_ are the main products from aerobic and anaerobic glucose degradation. They can be hydrolyzed into lactate/H^+^ and HCO_3_^−^/H^+^ respectively, both in the cell and outside the cell after passing through the plasma membrane in the form of an unhydrolyzed state. Lactate is excreted with facilitated transport by monocarboxylate carriers and anion exchange proteins [112]. Meanwhile HCO_3_^−^ can be transported outside the cell by an antiport. During the hydrolysis process, intracellular and extracellular protons are generated. The intracellular ones have to be transported outside by a Na^+^/H^+^ exchanger. Besides, when the cell receives external stimulation, e.g., exposure to a toxic agent or receptor activation, the metabolic activity or the ionic equilibrium in the cell can be interfered with, and the consumption of cellular ATP may change. Since ATP hydrolysis is closely related to the production of acidic metabolites, it can cause changes of extracellular acidification rate (ECAR).

As mentioned above, LAPS has many advantages for constructing cell-based biosensors. Since the first publication of the Cytosensor^TM^ Microphysiometer, it has been widely used. Several reviews [28,44,49,113,114] have been published to introduce the application of the microphysiometer. Besides, the newly proposed cell-semiconductor LAPS device for extracellular detection is considered as a useful tool for cell electric biology study. With the microphysiometer, functional characteristics of ligand/receptor can be studied, usually by monitoring the time-dependent and dose-dependent response of ligand/receptor binding. The time-dependent response shows the dynamic characteristics of ligand/receptor binding, while the dose-dependent response indicates the activation dose of the receptor.

The identification of specific and functional orphan receptors will facilitate studies on their physiological roles and the search for receptor agonists and antagonists. Due to the lack of a proper radioactive label, screening this type of ligand and receptor with radio receptor assay (RRA) is too difficult. It is also difficult to solve this problem with the biochemical methods, as the details in the mechanism of this type of receptor are not very clear. When using the microphysiometer, a generic characterization of ligand/receptor can be obtained. But it is usually insufficient for the identification of ligand/receptor. Therefore, other methods are usually used along with the microphysiometer to give precise identification of ligand/receptor. 

**Figure 19:**
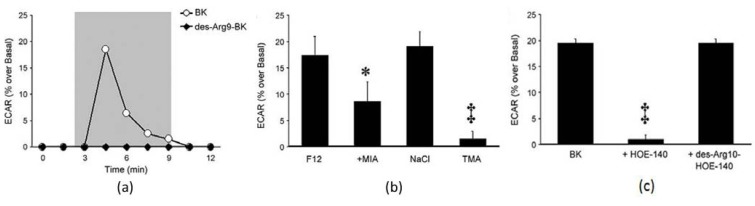
ECAR of HEK293 cells under stimulation of BK. (**a**) ECAR measurements were monitored. BK (white circles) stimulates ECAR, whereas the BK B_1_ receptor agonist des-Agr9-BK (dark circles) does not. Cells were exposed to perfusate-containing drug during the time span encompassed by the gray box; (**b**) ECAR stimulated by 10^−^^6^ M of BK in various buffers, including Ham’s F12 medium, without and with 10^−5^ M MIA, a balanced salt solution containing NaCl or TMA substituted mM per mM for sodium. * P < 0.05 *vs*. BK alone; zP < 0.01 *vs*. BK in balanced salt solution with NaCl; (**c**) Effects of BK B_2_ (HOE-140) and BK B_1_ (des-Arg^10^-HOE-140) receptor antagonists on BK-stimulated ECAR. Antagonists (10^−5^ M) were added 30 min prior to addition of BK. All experiments were performed at least 4 times. zP < 0.01 *vs*. BK alone. Error bars in (b) and (c) represent the S.E.M. (Reprinted from [115]. © 2009, with permission from Elsevier).

ECAR of CHO cells expressing orphan GPCRs was measured [116]. When treated with synthetic compounds, such as bioactive peptides, it was found that CHO cells expressing an orphan GPCR, FM-3 were responsible to neuromedin U in the microphysiometric assay. It was also discovered that bradykinin (BK) activated extracellular signal-regulated protein kinase 1 and 2 (ERK) in the human embryonic kidney (HEK) 293 cells [115]. ECAR was a reflection of the Na^+^/H^+^ exchange (NHE). When combined with intracellular Ca^2+^ concentration from fluorescent measurements, and ERK activation assessment through western blotting with a phosphor-specific ERK antibody, it was found that signals obtained by exposure of HEK 293 cells to BK, were blocked by HOE-140 (B_2_ receptor antagonist) but not by des-Arg^10^-HOE-140 (B_1_ receptor antagonist) (Figure 19). Thus it was concluded that HEK 293 cells express endogenous functional BK B_2_ receptors. 

### 3.4. Cellular Redox Potential Detection

Cell metabolism is accompanied by various oxidation-reduction reactions, such as hydrogen transfer reactions and electron transfer reactions in the respiratory chain. Maintaining the proper concentrations of oxidants and reductants may be essential for cell health. The excretion of acidic waste from intra-cellular fluid has been used as a parameter to evaluate the metabolism state of cells. Similar to the acid-base mechanism, the redox potential in tissue maintains within a narrow range and injury may be brought about at the cellular, subcellular, and molecular level when the balance breaches. The concept of redox potential balance is not new but has not received the attention of that associated with acid-base chemistry. One of the reasons may be the lack of effective and direct measuring technology for redox potential when involved in biological systems. 

The early measuring method is based on the dyes (such as mythylene blue) that indicate the redox potential through their color change [117]. The earliest application of redox potential for the extra-cellular phenomenon was the measurement of oxidation potential of *in vitro* plant oxidase using a gold electrode electrochemically. Then, electrodes (mainly platinum or gold electrode) had been used as the major effective way of redox potential detection in several decades. The cells and tissues measured included yeast, various bacteria [118,119,120], thin slices of liver and muscle suspended in buffer solutions, and tumors *in vivo* [121]. Until now the potentiometric measurement using noble electrodes has been widely used in redox potential monitoring of cell culture [122,123]. Usually, the redox potential electrode is worked as a working electrode and connected to a reference electrode through a potentiometer. Besides, Ward *et al.* [52] developed an universal redox electrode by modifying a Au wire electrode with electron transport promoter 4-pydidinethiol and bis(4-pyridyl)disulfide, which improved the performance of the electrode and avoided biofouling from biological samples. As the electrode technology and manufacturing process approaches maturity, commercial combined redox potential electrodes have come within our range of vision. The combined electrodes are mostly applied in biotechnology or fermentation because of their convenient usage in comparison with the traditional two-electrode system in electrochemical cells. 

In biotechnology and microbiology, redox potential is one of the most complex indicators of the physiological state of microbial cultures and its measurement is useful for qualitative and quantitative determination of the microbial contamination [124]. Besides, the significance of redox potential is not limited to functioning as an indicator to reveal related state about cultures, another no less important application is to externally control the redox potential to lead the system developing as premeditated. Zheng *et al.* [125] adopted extracellular oxidation-reduction potential (ORP) of −170 mV and obtained a maximal 176 g/L lactic acid production compared to other different ORPs. Koo *et al.* [126] found that the production of coenzyme Q_10_ can be improved by increasing the NADH/NAD^+^ ratio in agrobacterium tumefaciens, which is also an example of redox potential working as a control factor. The indicator and controller roles are just like the two sides of a coin, which also can be applied to cell-based research. 

For decades, the redox potential has become an important parameter monitored in cell culture medium. The study focuses on cell metabolism detection. Considering the complexity of redox potential in cell culture medium, other parameters like ATP, dissolved oxygen, glucose and glutamine consumption are also monitored to obtain a general analysis of cellular physiological states. Hwang and Sinskey [127] used the redox potential measurement of the medium using a platinum electrode and empirical correlation of the cell concentration with the redox potential for on-line estimation of viable cell concentration. It has been demonstrated that this method is applicable to various cell lines. Later, Eyer and Heinzle [122] used a method based on an ATP balance with ATP steady-state assumption to estimate viable cells in a hybridoma culture and confirmed the validity of viable cell estimation using redox potential measurement. Cell metabolism is always accompanied by substance consumption or depletion, which can be indicated by a decreasing redox potential. Higareda *et al.* [123] studied the relationship between culture redox potential (CRP) and glucose depletion in hybridoma cultures, and found that simultaneous measurement of oxygen uptake rate (OUR) and CRP can permit the discrimination between operational eventualities and real metabolic events. 

However, the measured redox potential of cell culture, also extracellular redox potential, is a comprehensive assessment of all redox pairs existing in the culture medium, and the measurement can be easily affected by pH and dissolved oxygen. Recent evidence suggests that the concentrations of specific redox couples may play a role in the regulation of other cellular functions, including gene expression. It is expected that the intracellular concentrations of redox couples like NAD^+^/NADH, NADP^+^/NADPH, CySS/CyS and GSSG/GSH may change selectively with changes in the cellular environment. Biochemical techniques exist for direct quantitation of most import cellular redox couples listed above, such as reverse-phase high performance liquid chromatography (rpHPLC) [128] or cellulose high performance thin-layer chromatography (HPTLC) [129], which have the ability to separate these species and realize measurement of specified species based on the reactivity of the thiol moiety, and a less specific detection method using membrane-permeable compounds which are fluorescent upon thiol conjugation [130]. Nevertheless, these biochemical techniques are invasive for cells and often based on endpoint detection. Electrochemical detection is expected to avoid the drawbacks and realize noninvasive and continuous monitoring of the intracellular redox state. An important difference between the electrochemical and biochemical methods is that the former measures the activity of cellular reactions rather than the steady-state concentration of a particular biochemical species. 

The first application of electrochemical-based biosensor for intracellular redox potential detection is reported by Rabinowitz *et al.* [51] who employed an EMIS structured sensor to probe intracellular redox activity in live cells. The sensor structure and measurement system adopted a modified Cytosensor microphysiometer. The electrochemical potential at the gold-electrolyte interface contributes to the reverse-bias potential acting across the photosensitive silicon semiconductor. Illumination of the silicon with a light-emitting diode generates an AC photocurrent in the external circuit, determined by the voltage applied through the controlling electrode. Changes in the redox potential at the gold surface are reflected through this current. In addition, there are no measurable direct current flows through the circuit during these measurements, which enables stable, rapid, low-noise measurements on microliter volumes. 

**Figure 20:**
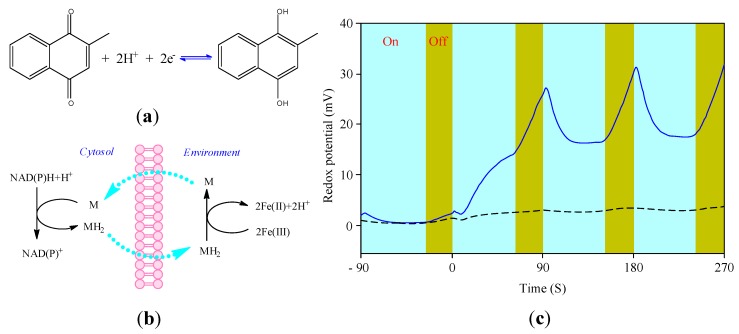
Detection of cellular redox reactions with use of menadione to link intra- and extra-cellular redox pairs. (**a**) Two-electron reduction of menadione to menadiol; (**b**) Hypothetical menadione reaction cycle; (**c**) Potentiometric measurements of menadione mediated ferricyanide reduction by cells. (Reproduced with permission from [51]. © 1998 American Chemical Society).

The most essential point in their study lies in measurement of the redox activity of the interior of cells noninvasively from extracellular microenvironment change. A carrier mediator that exchanges electrons rapidly with both the exterior and interior couple and links the extracellular electrolyte solution to intracellular redox pairs was used. As shown in Figure 20, the couple menadione/menadiol was used as an effective carrier mediator, because both of them are lipid soluble. Menadione and menadiol can be oxidized and/or reduced by intracellular enzymes. The major enzyme had been demonstrated to be NADPH, produced by the pentose phosphate pathway. In the meantime, the external medium bathing the cells contains a ferricyanide/ferrocyanide couple which reacts rapidly with menadiol. Thus the monitored redox potential of the ferricyanide/ferrocyanide couple is a measure of intracellular redox activity. Using this method they studied three cell lines (L929, CHO, and CH27), and the effect of reactions of mitochondrial electron transport chain and signal transduction cascade on the cell reduction rate. 

### 3.5. Cytotoxicity Assays and Drug Screening

#### 3.5.1. Drug Analysis Using MEA

Cardiomyocytes on MEA have been used in a number of studies to investigate the effect of cardioactive drugs and environmental toxins, such as pesticides [131] which affected the shape of field potentials recorded by MEA. 

Drugs can interfere with the normal operation of many ion channels, including sodium, potassium, and calcium ion channels that regulate heart activities. The fast inward Na^+^ current is responsible for the depolarization. When treated with Na^+^ channel blockers, the amplitude of extracellular field potential greatly decreases and the conduction delay increases [132,133,134]. Ca^2+^ is closely related to cardiac contractility. Drugs like quinidine and nifedipine can cause changes in the shape of extracellular field potentials and beating behavior [134,135]. The cardiotoxicity of different drugs of potassium channel openers, such as angiotensin II [136,137], is also studied. Generally, the drugs caused complicated effects on more than one specific type of ion channel and cellular organ. 

An important parameter that cause cardiac arrhythmia is the prolongation of the QT interval of the surface electrocardiogram (ECG), which corresponds to the duration of the field potential recorded by MEA [138]. The QT-Screen is a 96-channel device for high-throughput pharmacological screening, and each channel contains one microelectrode (Φ = 100 μm) [139]. It is specially designed to natively analyze the drug-induced QT-prolongation and cardiac arrhythmia *in vitro*. 

Neuronal network grown on MEA responds to neuroactive compound sensitively with changes in their native, spontaneous activity patterns. This altered activity is often substance- and concentration-specific. The influence includes direct metabolic effects, specific synaptic effects, transmission effects that stop AP propagation and generic mebrance effects mediated through non-synaptic Ca^2+^ or K^+^ channels or by the generation of new channels [140]. Some typical neuroactive substances with effects of excitation, inhibition or disinhibition have been studied [64,141,142,143,144,145,146,147,148,149,150], as shown in Table 1. 

**Table 1:** The study of pharmacology on neuronal networks based on MEAs.

Stimulus and concentration	Cellular component	Description	Reference
*N*-methyl-D-aspartate (NMDA) (10 μM)	Cortical neurons	NMDA drastically increased the bursting activity. The spike rates and frequencies were increased, but the pattern and shape regularity of bursting neural signal were decreased.	[141]
γ-aminobutyrate (GABA) (30 μM)	Mouse spinal nerve cells	The neural signal bursting was totally inhibited at 30 μM GABA.	[142]
Dopamine (15–100 μM)	Cortical neurons	Dopamine dispersed correlations between individual neuronal activities while preserving the global distribution of correlations at the network level.	[143]
APV (NMDA receptor antagonist) (40–100 μM)	Spinal cord networks	The rate of neural signal bursting was reduced by half.	[144]
Strychnine (0 nM–20 μM)	Spinal cord networks	Neural signal bursting was increased at 5–20 nM and coordinated above 5 μM.	[145]
GABA (10 μM), Bicuculline (10 μM)	Olfactory placode neurons	GABA almost immediately inhibited firing activities. Bicuculline had the facilitatory effect or lack of effect.	[151]
Eserine (10–150 μM)	Cortical cultures	The increase of bursting and spiking at 10 and 25 mM was concentration dependent, but it was inhibited at concentrations above 50 mM.	[147]
Ethanol (40 mM)	Embryonic murine neuronal networks	Spontaneously active frontal cortex cultures showed repeatable, concentration-dependent sensitivities to ethanol, with initial inhibition at 20 mM and a spike rate 50% effective concentration (EC_50_) of 48.8 ± 5.4 mM.	[148]
Tetrodotoxin (TTX, 100 μM)	Retina	The transient ON-response of ganglion cells was blocked by the sodium channel antagonist TTX.	[150]
Zn	Cortical neurons	The enhanced, coordinated bursting and spiking were increased, followed by irreversible activity decay.	[64]
Botulinum toxin (BoNT, 10 ng/mL)	Cortical networks	The duration and number of spikes were increased.	[149]

#### 3.5.2. Drug Analysis Using ECIS

Cell-based assays have been used as a substitutional method for animal experiments in pre-clinical research, development of drugs and toxicological testing [152,153,154]. It has been concluded that *in vitro* cytotoxicity assay can be used as an adjunct to animal testing to improve dose level selection and thus further reduce the number of animals used [155]. ECIS has been proven valuable and reliable in real-time monitoring of dynamic changes induced by cell–toxicant interactions [156]. 

**Figure 21:**
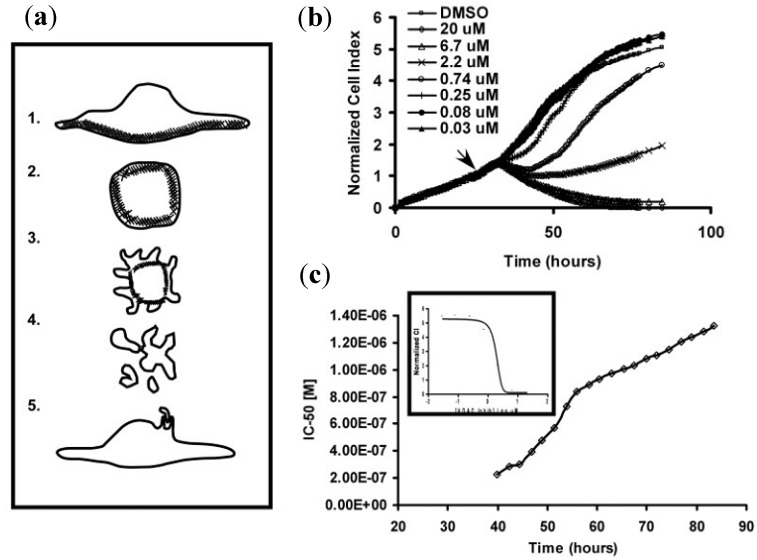
(**a**) Morphological changes associated with apoptosis. (1) shows a cell under normal physiological conditions. The apoptotic response leads to cellrounding (2) followed by membrane blebbing, which occurs due to weak interaction between the membrane and actin cytoskeleton (3), formation of apoptotic bodies (4), and ultimate actin-mediated engulfment of apoptotic bodies by neighboring cells (5); **(b)** Dynamic monitoring of cytotoxicity using the RT-CES system. A549 cells were seeded in microtiter plates containing interdigitated microelectrodes and treated with the HDAC inhibitor Scriptaid at the indicated doses; (**c**) Real-time IC_50_ values for Scriptaid. The inset shows the dose-response to Scriptaid at a single time point. (Reprinted from [165]. © 2008, with permission from John Wiley and Sons).

Cells respond to cytotoxins in the form of loss of adhesion and cell rounding, membrane protrusions or blebbing, formation of apoptotic bodies, and the ultimate engulfment of apoptotic bodies induced by phagocytosis. These apoptotic responses indicate changes in cell adhesion and morphology, which will ultimately induce a decline of cell-substrate impedance in an acute or chronic manner. Such nonlinear dynamic changes depend largely on cell types, compound properties and concentration, and compound exposure duration (Figure 21). Keese *et al.* [157] demonstrated the applicability of the ECIS system for toxicological testing of fibroblasts and epithelial cells. Subsequently, many chemicals, such as antipyrine, trichlorfon, dimethyl formamide and sodium dichromate, as well as some familiar toxins such as sodium arsenite (As(III)), mercury (II) chloride, benzalkonium chloride (BAK), triton X-100, sodium lauryl sulfate, cadmium chloride (CdCl_2_), 1,3,5-trinitrobenzene (TNB), cycloheximide (CHX), and neutral red solution have been tested for cytotoxicity evaluation with various types of cells [156,158,159]. The effect of cytotoxins on cells is usually presented as time-response and dose-response curves using ECIS technology in a label-free way. 

Conventional cell-based assays are often labor-intensive, non-automatic and time-consuming. These major drawbacks are more prominent in drug testing. With the advantages of real-time monitoring and easy operation, ECIS comes to be especially suitable for cell-based HTS. Some corporations have launched their own products for HTS based on ECIS, such as ACEA Biotech Inc., Applied Biophysics Inc., Bionas Inc., and Molecular Devices Inc. In addition to HTS, measurement of an increasing number of cellular parameters in parallel is another promising method in screening applications as a comprehensive view of the investigated cellular process can be obtained [160,161,162,163,164]. 

However, the impedance measurement of a large population of cells attached on one electrode may result in complex and even questionable interpretations, because only an overall level of cellular activities can be detected without differentiating the effect of proliferation, motility, and cell-cell separation. It is difficult to precisely monitor changes in membrane properties of individual cells with the current ECIS systems. The response of a single cell to a given dose of a pharmacological drug or harmful toxin may be more significant than that of multiple cells. In a single-cell sensing system, cells respond to drugs individually, and this effect can be accurately recorded without cell-cell interactions [166,167].

#### 3.5.3. Drug Analysis Using LAPS

By monitoring extracellular parameters after drug treatment, the corresponding drug effect can be directly investigated. Both the microphysiometer and the cell-semiconductor hybrid LAPS device can be used for drug analysis and evaluation, depending on the extracellular parameters to be measured. The microphysiometer has become a utility tool in drug analysis, screening of prospective pharmacological agents, characterizations of dose responses and structure-activity relationships, and investigation of cellular mechanisms. 

**Figure 22:**
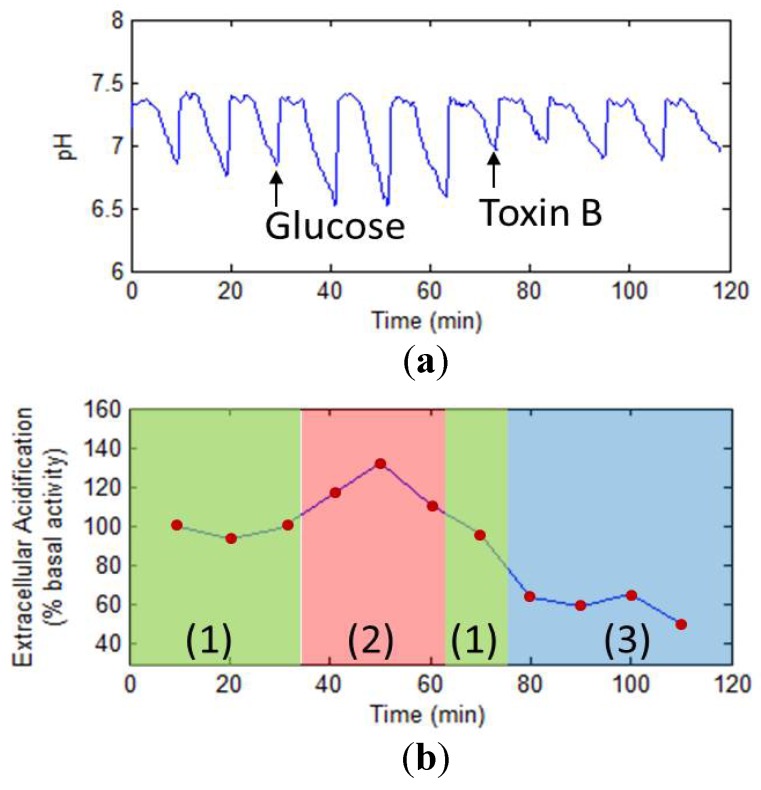
Cellular metabolism detection under drug stimulation. (**a**) Original data shows the change of pH corresponding to the photocurrent; (**b**) The extracellular acidification rate under the blank (1), glucose (2) and clostridium difficile toxin B (3). (Reprinted from [111]. © 2012, with permission from World Scientific Publishing Co.)

In the work of Hu *et al.* [111], the metabolic activities of MCF-7 cells were monitored by the secretion of the cellular acidic metabolites. The extracellular acidification rate was one of the most significant parameters which indicated cellular metabolic rate. Therefore, the extracellular acidification was monitored to determine the performance of the microphysiometer. Firstly, the extracellular acidification rate was recorded in the presence of cell culture medium alone as basal extracellular acidification rate. Subsequently, glucose and clostridium difficile toxin B was injected respectively into the sensor unit, and the change of extracellular acidification was monitored. Figure 22 displays the cell responses in the stage of blank (1), glucose (2), and clostridium difficile toxin B (3). In the absence of drug, the cells secreted the acidic metabolites in the native state. Meanwhile, the glucose induced a sharp increase in the extracellular acidification rate of MCF-7 cells, with a maximum about 138% after 40 min. Then, clostridium difficile toxin B was injected into the culture medium and induced a 40% decrease of the basal extracellular acidification rate after 70 min. 

Glucose is the necessary nutrition and supplies the main energy ATP in normal cellular life. With the increase of glucose, intake of glucose was sharply raised by MCF-7 cells, which increased the generation of the protons under aerobic and anaerobic conditions. Therefore, after the cells absorbed more glucose, metabolic activities were enhanced in the form of an increased extracellular acidification rate. Meanwhile, clostridium difficile toxin B is a toxin generated by clostridium difficile. It is usually described as an enterotoxin, but it also has some activities as a cytotoxin [168]. Toxin B is a chromosomally-encoded exotoxin that is produced and secreted by several bacterial organisms. It is often heat-stable, is of low molecular weight and water-soluble. Enterotoxins are generally cytotoxic and kill cells by altering the apical membrane permeability of the epithelial cells. The action of enterotoxins increased chloride ion permeability of the apical membrane of cells. These membrane pores were activated either by the increased cAMP or by increased intracellular calcium ion concentration. Therefore, the toxin B affected the normal metabolic activities of MCF-7 cells and induced a weak extracellular acidification rate. 

Multifunction is also essential for basic research as well as for various fields of biomedical applications. The multianalyte function for extracellular ion monitoring, such as Na^+^, K^+^, Ca^2+^, may change along with cellular metabolism. In order to analyze simultaneously the relations of the extracellular environmental H^+^, Na^+^, K^+^, Ca^2+^ under the effects of drugs, a novel microphysiometer was developed based on multiparameter-LAPS [29,169]. 

**Figure 23:**
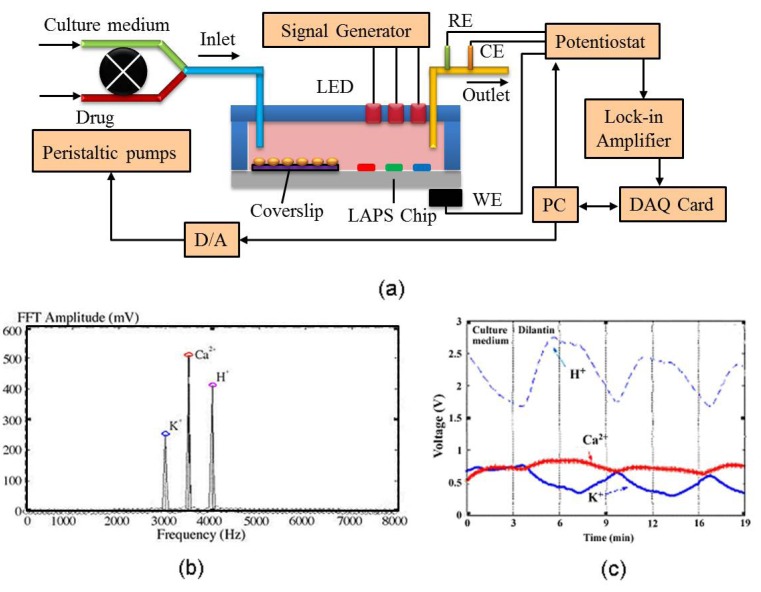
Multianalyte microphysiometer studies based on LAPS. (**a**) The schematic drawing of the multiparameter-LAPS system to different extracellular ions (H^+^, K^+^, and Ca^2+^); (**b**) FFT analysis of signals from three sensitive membranes with three light sources; (**c**) Simultaneous analyzing of H^+^, K^+^, Ca^2+^ by multi-LAPS. (Reprinted from [29]. © 2001, with permission from Elsevier).

The LAPS surface was deposited with different sensitive membranes by the silicon microfabrication technique and the PVC membrane technique. The different sensitive membranes (H^+^, K^+^, Ca^2+^) are illuminated in parallel with light sources at different frequencies (3 kHz for K^+^, 3.5 kHz for Ca^2+^, 4 kHz for H^+^) (Figure 23(a)). The amplitude of each frequency component might be measured online by software FFT analysis (Figure 23(b)). Dilantin, *i.e.*, phenytoin sodium, a sort of anti-epilepsy drug and anti-arrhythmia drug, had significant effects as a tranquilizer, hypnotic and anti-seizure agent. It was proved that dilantin had membrane stabilizing action on neural cells because it reduced the permeability of pericellular membrane ions (Na^+^, Ca^2+^), inhibited Na^+^ and Ca^2+^ influx and staved K^+^ efflux. Thus, the refractory period were prolonged, pericellular membrane stabilized and excitability decreased (Figure 23(c)). 

## 4. Development Trends of Electrochemical Cell-Based Biosensors

### 4.1. Integrated Sensors for Multi-Parameters Monitoring

Cellular life is characterized by dynamic molecular processes whose highly interconnected regulation is essential for the whole organism. Cells continuously integrate different sources of chemical and physical signals originating from both internal and external environments. The ‘output’ of this cellular signaling network may be manifested as a decision about growth and mitosis, activation of distinct metabolic pathways, the production and release of proteins or the initiation of programmed cell death. Conventional methods, for instance fluorescence techniques, are most often used to dissect the molecular basis of cell functions, either with fluorescent protein analogs or with synthetic dyes. However, no truly appropriate fluorescence techniques have been developed so far for the analysis of cell electric activity, morphology and metabolic rates. Functional cellular assays are employed to monitor several kinds of signals at the same time. Thus the signal-processing of living cells can be profiled in a more detailed and thorough way.

**Figure 24:**
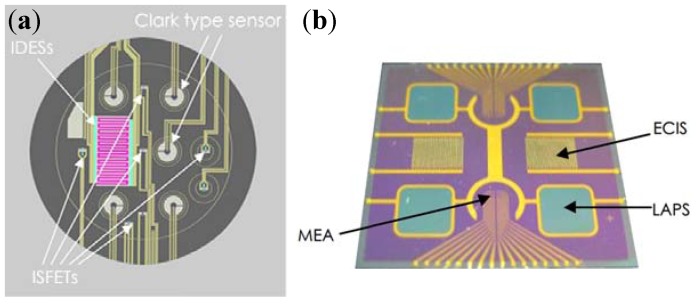
(**a**) A metabolic chip integrated with IDES, ISFET sensors and Clark-type electrodes. (Reprinted from [163]. © 2007, with permission from Elsevier). (**b**) Integrated biochip with MEA, ECIS and LAPS.

Brischwein *et al.* [160] first reported on multi-parametric cell based assays with data obtained solely with integrated sensors on silicon chips. Extracellular acidification rates (with ISFETs), cellular oxygen consumption rates (with amperometric electrode structures) and cell morphological alterations (with impedimetric electrode structures, IDES) were monitored simultaneously for up to several days. Ceriotti *et al.* [163] employed a multi-parametric chip-based system to measure cell adhesion, metabolism, and response to metal compounds which was previously classified as cytotoxic in immortalized mouse fibroblasts (BALB/3T3 cell line). In a parallel, online, and in a label-free way, the system measured extracellular acidification rates (with ISFETs), the cellular oxygen consumption (with amperometric electrode structures (Clark-type sensors)), and cell adhesion (with IDESs) (Figure 24(a)). A group from the biosensor national special lab in China has established an automatic multi-parametric platform based on an integrated biochip which measures the cell electric activity (with MEA), the cell morphology (with ECIS) and metabolic rates (with LAPS) (Figure 24(b)). Using the integrated multi-parametric sensors, some cell-based assays are conducted, including cytotoxicity and drug assessment, cell development and stem cell differentiation research. 

**Figure 25:**
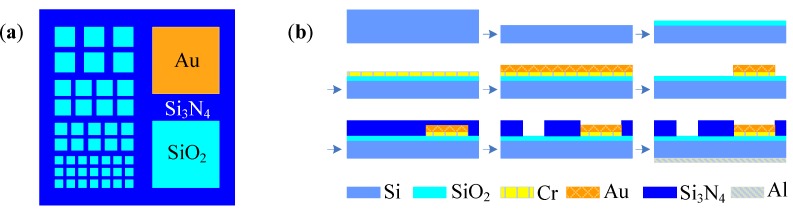
Schematic diagram of an integrated photovoltage-based biosensor used for cellular detection. (**a**) Sensor design; (**b**) Sensor fabrication.

From another point of view, extracellular acidification and redox potential are important indicators of cell metabolism activity. As the EIS and EMIS sensor have similar structure and working principle, it makes sense to integrate the two sensors on the same chip. The related work has been carried out by Wang *et al.* [170]. The integrated potentiometric sensor (Figure 25) was constructed by depositing a gold metal layer on a partial surface of silicon dioxide. The sensitivity for pH and redox potential measurement is approximately 41.6 mV/pH and 53.2 mV/log[Fe(III)/Fe(II)], respectively. The latter is in accordance with the work of Adami *et al.* [171]. The integrated sensor was used for nephrotoxicity assay under drug stimulation. 

The physiological state shows an acidified extracellular microenvironment and a reduced redox potential. It is revealed from this study that gentamicin inhibits the regular cellular metabolism, and the toxicity enhances with the concentration, but the toxicity is reversible when washed with PBS solution, which is in accordance with reversible or partially reversible kidney failure induced by gentamicin in clinical use, while 5-FU exhibits an anti-metabolism effect both intra and post 5-FU administration. The change of extracellular redox potential is significantly different from that of acidification, which indicates that the drugs are toxic for kidney cells mainly through acidification inhibition but induce no significant change to the concentration of extracellular redox pairs. However, this study was just a preliminary application of integrated photovoltage-based biosensors for cellular toxicity evaluation and a complete dose-response analysis and EC_50_ determination was not presented. Anyhow, the concept of an integrated sensor for acidification and redox potential detection has potential in drug toxicity analysis and can work as a supplementary means for preclinical drug screening. 

### 4.2. Cell-Based Electrochemical Biosensor with Microfluidic Technology

Microfluidic technology is a powerful tool to facilitate the miniaturization and integration of analysis systems and can even address many issues that traditional methods cannot deal with. It has a cell-comparable small size and can precisely confine cells in the specific microchannel. By employing micro pumps and valves, mechanical or chemical stimulus can be introduced to cells and the corresponding cellular level biological responses can be monitored by integrated electrochemical electrodes. Even single cell and small cell group analysis can be achieved. Besides, microfluidics can realize high-throughput parallel biological monitoring and analysis.

**Figure 26:**
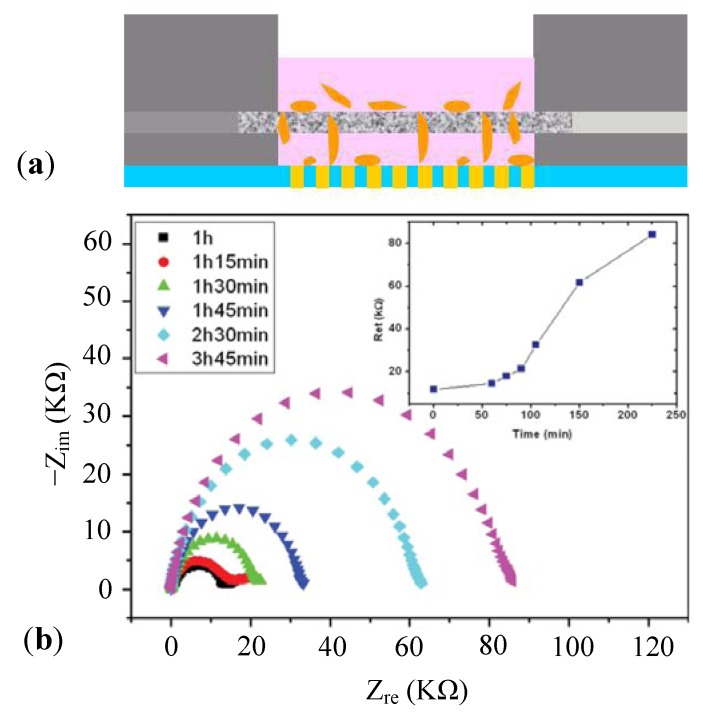
(**a**) Autotranswell cell chip for migration assay; (**b**) Real time monitoring of cell migration. Inset shows the time course of transmigration.

The microfluidic chip with specific micro-fabricated electrodes has broad applications in cell morphology research and has many dramatic advantages over traditional assays, such as simple operation, rapid detection, and less invasiveness. Cells are cultured in the microfluidic chip and the culture media solution can be introduced automatically through the microfluidic channel. Using suitable electrochemical detection methods, cell morphology information, such as adhesion, migration, proliferation, and apoptosis, can be monitored in real time. One typical example is the automatic transwell assay, in which two microfluidic chambers separated by a porous membrane are designed to monitor cell migration [172]. As illustrated in Figure 26(a), cells are seeded in the upper chamber through microfluidic channels and an EIS sensor is placed in the lower chamber. Once cells migrate from the upper chamber into the lower one, great impedance changes can be detected. Figure 26(b) shows the impedance changes at different times. In addition, the influence of different ECM components can be also detected by this assay. 

The microfluidic channel also provides great convenience for cellular metabolism analysis, especially in terms of single cells and small cell groups. Cells can be cultured in a tiny space and their physiological and electrical responses will be captured easily. Currently, many researches are covered in analyzing extracellular microenvironment [173] and intracellular components [174], monitoring ligand-induced cell secretion [175], recording the extracellular potential [176] and cell dynamic responses [177,178], *etc*. Besides, the cell patterning technique is an important issue in fundamental cell biology, tissue engineering, and neuron network research. Microfluidic technology plays a great role in this field. The chemical and cellular environment can be controlled precisely through the microchannels modified with diverse chemical molecules [179,180,181]. 

Assisted with microfluidic technology, cell-based electrochemical biosensors will have broad commercial and medical applications in the future. Low cost, disposable chips can be produced in large amounts and point-of-care diagnosis can be realized in the near future. 

### 4.3. Cell-Based Electrochemical Biosensor with Nanotechnology

In recent years, nanotechnology has been greatly improved and widely applied in the biosensing fields. The most commonly used nanomaterials include nanoparticles, nanotubes, nanowires, and nanobars. Due to attractive electronic, optical, magnetic, thermal and catalytic properties, they have important applications in many interdisciplinary fields. 

**Figure 27:**
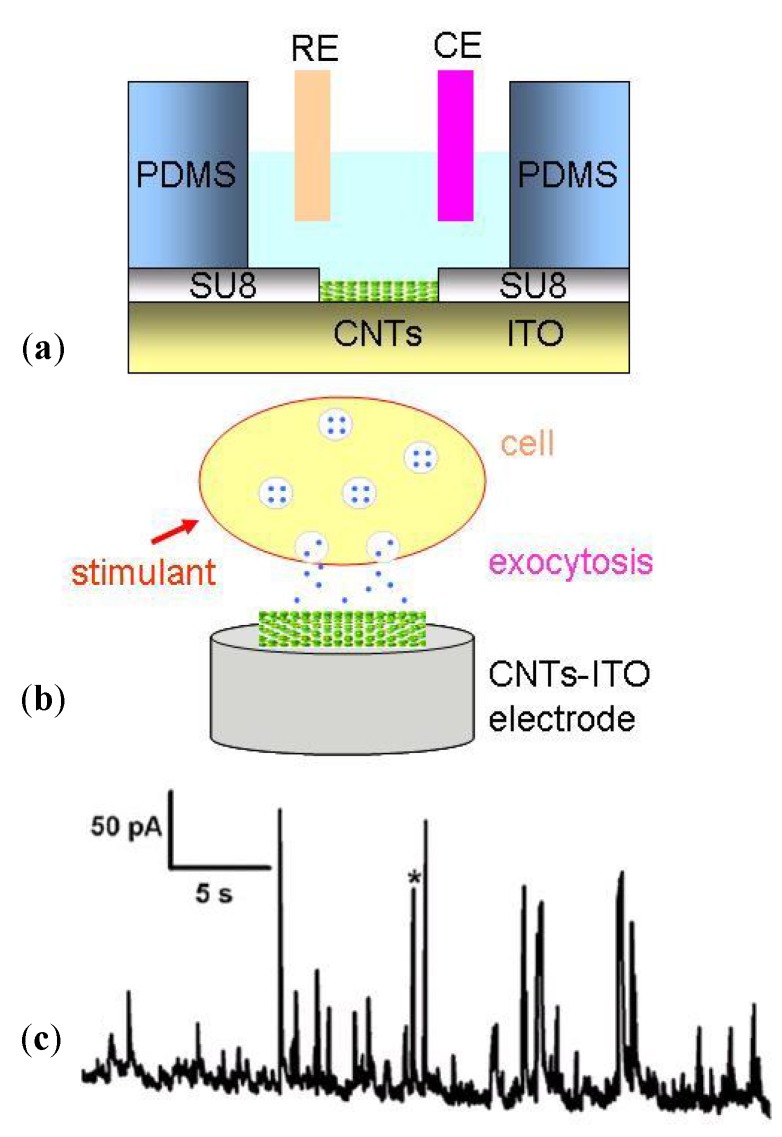
(**a**) Cross-sectional schematic of the electrochemical sensor with CNTs-ITO microelectrode. Ag/AgCl acted as reference electrode (RE) and Pt wire as counter electrode (CE); (**b**) Schematic of the single-cell release monitoring with the CNTs modified ITO electrode; (**c**) The amperometric response to stimulation from a single cell. (Reprinted from [183]. © 2011, with permission from Elsevier).

Nanomaterials play important roles in many aspects of biosensors. Owing to the large surface-volume ratio, nanogold particles are employed to modify the sensitive surface and enlarge the effective working area. Magnetic nanoparticles are usually used to accumulate or separate some important biomolecules conveniently [182]. Since carbon nanotubes (CNTs) have high electrocatalytic effect and fast electro-transfer rate, they are always used in the detection strategy to assist signal detection [183]. As shown in Figure 27(a), an indium tin oxide (ITO) electrode modified with CNTs was used as the working electrode and SU8 was patterned to limit the dimension of the ITO electrode. PC12 cells were cultured in a limited chamber (hundreds of μm^2^). Once extracellular stimuli induced the exocytosis of cells, the molecules can be detected by the CNTs-ITO electrode, as illustrated in Figure 27(b). The results demonstrated that the sensitivity of the electrochemical sensor after CNTs surface modification was improved by 2.5–3 orders of magnitude and a typical amperometric response from a single cell was shown in Figure 27(c). The distinct optical performance of nanomaterials is also very attractive in the development of optical biosensors. Many kinds of nanomaterial-based biosensors are investigated to detect DNA, protein, and cellular substance by diverse detection technologies [184].

As an attractive trend, the distinct performance of nanomaterials can contribute much to cellular diagnosis. Nano-dimension electrodes can be used to probe intracellular compounds and monitor cell metabolism and electrophysiological activities. Nanomaterials can help to simplify and miniaturize biosensors for biomolecular trace detection. 

## 5. Conclusions

This review essentially presents the microfabricated electrochemical cell-based biosensors that are commonly used in living cell analysis. MEA is applicable mostly for the electrophysiological recording of electrically excitable cells, and analysis of signal transduction between different cells. ECIS has become mature in the morphology monitoring of anchorage-dependent cells. LAPS is outstanding in the metabolite detection of the extracellular microenvironment. These microfabricated biosensors have demonstrated good performance in the mechanism study of cellular level, and even in drug analysis and environment monitoring. The biggest challenge in the development of high-precision commercialized cell-based biosensors is the designated growth and strong adhesion between the chip and cells. The introduction of nanotechnology and microfluidic technology may help to improve the performance of biosensor systems mentioned above and accelerate the birth of novel biosensors. It can be expected that the entrapment of cells and construction of single-cell-based biosensors will be facilitated. Another important development is high throughput and multi-parameter detection. Investigations of multi-channel and multi-functional integrated biosensors are gradually carried out, and this will result in a higher demand for supporting measurement systems. 
